# Nodular Histiocytic/Mesothelial Hyperplasia Mimicking Mesenteric Metastasis

**DOI:** 10.7759/cureus.24971

**Published:** 2022-05-13

**Authors:** Joseph Grech, Cullen M Lilley, Emily M Martinbianco, Xianzhong Ding, Kamran M Mirza, Xiuxu Chen

**Affiliations:** 1 Department of Pathology, Loyola University Chicago Stritch School of Medicine, Maywood, USA; 2 Department of Pathology and Laboratory Medicine, Loyola University Medical Center, Maywood, USA

**Keywords:** histiocytosis with raisinoid nuclei (hrn), intralymphatic histiocytosis (ilh), histioeosinophilic granuloma (heg), reactive eosinophilic pleuritis (rep), mesothelial/monocytic incidental cardiac excrescences (mice), nodular histiocytic/mesothelial hyperplasia (nhmh)

## Abstract

Nodular histiocytic/mesothelial hyperplasia (NHMH) is a rare histologic entity, characterized by localized benign reactive proliferation of histiocytes and mesothelial cells. The presence of this rare entity poses a challenge in differential diagnosis, both in radiological findings and pathological interpretations under certain circumstances, and consequently has been misdiagnosed as malignancy. Here, we report a case of mesenteric NHMH in a patient with colonic mucinous adenocarcinoma. Histology shows numerous large calretinin (+) mesothelial cells mixed with CD68 (+) histiocytes by immunohistochemistry. In contrast to almost all previously reported cases with typical features of histiocytic predominance, the current case of NHMH mainly consists of mesothelial cells with intermixed histiocytes. The findings expand the histologic spectrum of NHMH and contribute to awareness of this entity in the differential diagnosis.

## Introduction

Nodular histiocytic/mesothelial hyperplasia (NHMH) is a rare benign tumor-like lesion characterized histologically by the localized reactive proliferation of histiocytes and mesothelial cells [1]. This lesion may occur at various sites of the body, predominantly mesothelium-lined locations, such as pleura, pericardium, and peritoneum, and rarely non-mesothelium-lined locations such as the endocardium and aortic dissecting aneurysms [2-4]. Less than 100 cases have been reported in the literature thus far. The main clinical significance for this entity is the challenging differential diagnosis under circumstances, such as metastatic adenocarcinoma, clear cell carcinoma of the lung, primary mesothelioma, Rosai-Dorfman disease, serous carcinoma, and Langerhans cell histiocytosis of the pleura, pericardium, cardiac valves, peritoneum, pelvis, and groin [2,5-7]. Here, we report a case of mesenteric NHMH in a patient with colonic adenocarcinoma clinically mimicking mesenteric metastasis. Histology shows numerous large calretinin (+) mesothelial cells mixed with CD68 (+) histiocytes by immunohistochemistry. In contrast to almost all previously reported cases with typical features of histiocytic predominance [1-2,4-5,7-9], the current case of NHMH mainly consists of mesothelial cells with scattered histiocytes. The findings expand the histologic spectrum of NHMH and raise awareness of histologic variation in this rare entity.

## Case presentation

**Figure 1:**
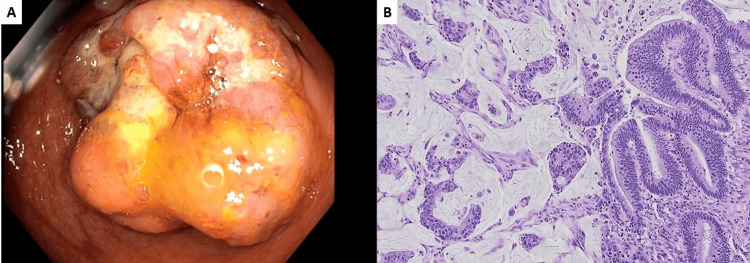
Cecal mass on endoscopy and microscopy Endoscopy shows a 5.0 cm circumferential and partially obstructing mass in the cecum (A). Microscopic examination confirmed the diagnosis of stage pT3N0Mx mucinous adenocarcinoma (B, 200x).

An 87-year-old male with a past medical history of hypertension and hyperlipidemia presented to the hospital due to scalp laceration and cerebral concussion following a fall at home. On admission, a workup showed he was anemic (Hgb 7.0 g/dL), and a positive stool guaiac test was noted. A computerized tomography scan with IV contrast showed a 5.5 cm large mass in the right colon with a severely narrowing colonic lumen. MRI reported a cecal mass and multiple subcentimeter mesenteric nodules suspicious for lymph node metastases or carcinomatosis. On colonoscopy, there was a 5.0 cm large, circumferential, fungating, and partially obstructing mass in the cecum (Figure 1A). Right hemicolectomy and omentectomy were performed to remove the cecal mass and regional lymph nodes. The tumor was pathologically staged as mucinous adenocarcinoma, pT3N0Mx (Figure 1B).

**Figure 2:**
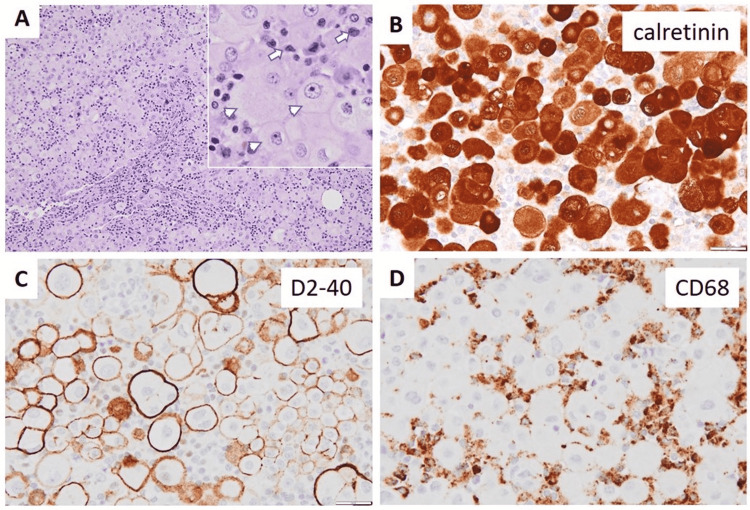
Morphologic features of mesenteric nodular histiocytic mesothelial hyperplasia (NHMH) on representative section The mesenteric nodular lesions show predominantly histiocytes (inset: arrows) and mesothelial cells (inset: arrowheads) as well as lymphocytes within the delicate fibrous septa by H&E stains (A). Immunostains confirm the presence of mesothelial cells with positive calretinin (B) and D2-40 (C), as well as histiocytes with strongly positive CD68 (D). Note: A 100x, B-D 600x

In addition to the adenocarcinoma in the cecum, the mesentery is focally thickened with small nodular lesions. Histology shows sheets of large round or polygonal cells with abundant pale finely granular cytoplasm, distinct cytoplasmic border, round or ovoid nuclei with prominent nuclear membrane, vesicular chromatin, single or multiple conspicuous nucleoli, and frequent binucleation in some cells. Rare mitosis is present. Prominent histiocytes with raisinoid nuclei are also present among the aforementioned large cells, and infiltrating lymphocytes are seen in focal fibrous septa. Immunostains show the predominant large cells are mesothelial cells (strongly positive for calretinin and D2-40) mixed with histiocytes (strongly positive for CD68) (Figure 2). Immunostains for CD1a, S-100, and CD34 are negative (not shown). Findings are consistent with the diagnosis of mesenteric NHMH.

## Discussion

In 1975, Rosai J and Dehner LP first described 13 cases of peculiar benign mesothelial hyperplastic lesions occurring during herniorrhaphy that closely resembled neoplastic processes, and they named this lesion "nodular mesothelial hyperplasia" based on morphologic features in absence of immunohistochemical study [10]. With the help of immunohistochemical analyses, it was later found that such lesions were composed primarily of histiocytes, and the entity was subsequently re-named “nodular histiocytic/mesothelial hyperplasia (NHMH)” by Chan JK in 1997 [5]. NHMH has since been found, most often incidentally, in various anatomical sites throughout the peritoneum and beyond, causing unnecessary anxiety and ancillary studies as well as exploratory surgical procedures.

To our knowledge, our case is the first case of NHMH found in the mesentery related to colon adenocarcinoma; however, there are many other medical conditions associated with NHMH (Table 1). Histologically, 72 out of 74 (72/74) cases reported in literature showed histiocyte predomination (Table 1). Consistent with this finding, Ordonez NG recommended using "nodular histiocytic hyperplasia (NHH)" to reflect the predominant cellular component of histiocytes in these lesions in 1998, instead of the terminology of NHMH [11]. One case reported by Bejarano PA showed a similar number of histiocytes and mesothelial cells [12], in contrast, our case exhibits a mesothelial cell predominance instead of histiocytes. Michal M compared the histologic features and immunoprofiles of 26 cases of NHMH, four cases of reactive eosinophilic pleuritis (REP), seven cases of mesothelial/monocytic incidental cardiac excrescences (MICE), four cases of histioeosinophilic granuloma (HEG), and nine cases of intralymphatic histiocytosis (ILH), concluding that these five lesions are variants of the same entity under a process of reactive histiocytic hyperplasia. Given the histiocytes in all these lesions exhibited typical cytologic features, raisinoid or reniform nuclei, and a moderate amount of finely granular pale or eosinophilic cytoplasm, the authors proposed a new comprehensive term "histiocytosis with raisinoid nuclei" [7]. Based on our experience, we believe the terminology of NHMH is more accurate since it includes both histiocytic and mesothelial cell populations, particularly for cases that are predominantly mesothelial cells such as our case.

**Table 1:** Summary of 74 NHMH cases reported in the literature ^a^ This case reported a similar number of histiocytes and mesothelial cells in the lesion [12]. ^b^ This is the current case, which is mesothelial cell-predominant in the lesion. NHMH: nodular histiocytic/mesothelial hyperplasia

Ref	Age	Sex	Clinical history	Site	Gross	Microscopy
[5]	57	m	Rickettsial pneumonia	Lung	Transbronchial biopsy	Histiocyte-predominant
[8]	71	m	n/a	Relapsed Inguinal hernia	Two nodules, 2.1 mm, 2.25 mm	Histiocyte-predominant
[2]	36	f	Abortion	Pelvis	Solitary, fine, filmy adhesion band on the ovary	Histiocyte-predominant
[2]	37	f	Abortions, endometriosis	Pelvis	0.5 cm endometriotic implants, a solitary, firm filmy adhesion on ovary and fallopian tube	Histiocyte-predominant
[2]	elderly	m	Lung carcinoma	Lung	Transbronchial biopsy	Histiocyte-predominant
[2]	53	f	Smoker, lung adenocarcinoma and carcinoid	Lung	Transbronchial biopsy followed by wedge resections	Histiocyte-predominant
[2]	47	f	Seasonal allergy, pericardial cyst	Pericardium	6.0 cm lesion in the right cardiophrenic angle	Histiocyte-predominant
[12]^a^	46	m	Heart transplant, bilateral lung infiltrates	Lung	Transbronchial biopsy	A similar number of histiocytes and mesothelial cells
[16]	25	f	Rheumatic pericarditis	pericardium	1.2 cm free-floating vegetation	Histiocyte-predominant
[3]	60	m	Coronary artery disease	Pericardium	1.5 cm cyst with nodules	Histiocyte-predominant
[4]	1.5	m	Spermatocele	Left groin	Solitary cyst with 0.5 cm mural nodule	Histiocyte-predominant
[4]	4.0	m	Spermatocele	Right paratesticular mass	3 mural nodules, 0.1-0.3 cm	Histiocyte-predominant
[4]	3.0	m	Inguinal hernia	Right groin	2 mural nodules, 0.2-0.4 cm	Histiocyte-predominant
[4]	2.5	m	Spermatocele	Left groin	Mural nodule and thickened cystic wall, 0.2-0.3 cm	Histiocyte-predominant
[4]	2.0	m	Inguinal hernia	Right groin	Single mural nodule, 0.2 cm	Histiocyte-predominant
[4]	5.0	m	Spermatocele	Right groin	Single mural nodule, 0.3 cm	Histiocyte-predominant
[4]	2.0	m	Spermatocele	Left groin	Cystic cotton-like material, focally thickened cystic wall, and vague nodule	Histiocyte-predominant
[9]	57	m	Subphrenic abscess, pleural effusion	Right parietal and diaphragmatic pleura	Multiple nodular lesions	Histiocyte-predominant
[7]	4-85 (mean 50.1)	15m, 32f, 3na	Peritoneal cyst, endometriosis, mesothelioma, ovarian cysts, ovarian serous carcinoma, solitary fibrous tumor, aortic dissection, pneumothorax, fluidothorax, goiter, myasthenia, thymoma, etc.	Peritoneum, pleura, pericardium, aorta, thyroid, thymus, fallopian tube, skin, etc.	From microscopic foci to 1-2 cm nodules	Histiocyte-predominant with various amounts of mesothelial cells
[11]	23	f	Metastatic sarcoma in lung	Right pleura	3.5 cm pleural cystic lesion	Histiocyte-predominant
[11]	78	f	n/a	Pleura	Pleural biopsy	Histiocyte-predominant
[11]	2	m	Inguinal hernia	Right groin	Small lesion	Histiocyte-predominant
[11]	74	m	Papillary urothelial carcinoma	Bladder wall	Bladder biopsy	Histiocyte-predominant
[1]	4	m	Spermatocele	Right groin	3.0 cm cyst with 0.6 cm mural nodule	Histiocyte-predominant
^b^	87	m	Cecal adenocarcinoma	Mesentery	0.5 cm focal nodular lesion	Mesothelial cell-predominant

Mechanistically, there are two hypotheses to explain the formation of these lesions. One is reactive hyperplasia. It has been postulated that NHMH occurs as a non-specific localized reaction to irritations such as trauma, tumor, or inflammation that leads to the aggregation of histiocytes and interaction with mesothelial cells [5,7-8,13-14], which is consistent with our case of colon adenocarcinoma. The "artifact theory" was proposed as a counterpoint to the aforementioned hypothesis and describes a mechanism by which the mesothelial and histiocytic cells were seeded either during a prior procedure or during biopsy [13,15]. To date, there is insufficient evidence to exclude either, and thus, both theories may co-exist until we gain further understanding of these lesions. In this regard, future molecular studies on involved histiocytes and mesothelial cells might be helpful to better elucidate the nature of NHMH and the relationship among the aforementioned lesions in different locations.

## Conclusions

NHMH is a rare entity that is most commonly encountered as an incidental finding. The main clinical significance of NHMH is its differential diagnosis with primary or metastatic diseases. It is important to be aware of its existence at different sites and histologic variations. An ancillary study to confirm biphasic cellular components of histiocytes and mesothelial cells may be necessary for challenging cases such as staging biopsies.
